# Implementation of an ERAS^®^ Programme for Total Hip and Knee Arthroplasty in a High-Volume University Hospital

**DOI:** 10.3390/jcm15020836

**Published:** 2026-01-20

**Authors:** Eric Albrecht, Marcio Oliveira, Valérie Addor, Julien Wegrzyn

**Affiliations:** 1 Department of Anaesthesia, University Hospital of Lausanne and University of Lausanne, Rue du Bugnon 46, 1011 Lausanne, Switzerland; 2 Department of Orthopaedic Surgery and Trauma, University Hospital of Lausanne, Rue du Bugnon 46, 1011 Lausanne, Switzerland; marcio.oliveira@chuv.ch; 3 Department of Development and External Affairs, University Hospital of Lausanne, Rue du Bugnon 46, 1011 Lausanne, Switzerland; valerie.addor@chuv.ch; 4 Department of Orthopaedic Surgery and Trauma, University Hospital of Lausanne and University of Lausanne, Rue du Bugnon 46, 1011 Lausanne, Switzerland; julien.wegrzyn@chuv.ch

**Keywords:** hip arthroplasty, knee arthroplasty, ERAS, analgesia, clinical pathway

## Abstract

**Background**. Enhanced Recovery After Surgery (ERAS) is a structured, multidisciplinary programme designed to optimise the entire perioperative pathway through evidence-based, patient-centred, standardised care. The objective of this report is to determine whether it is feasible to implement an ERAS^®^ program in orthopaedic surgery within our institution for primary THA and TKA. **Methods**. This single-centre quality-improvement project followed the ERAS^®^ Society certification framework to create and implement an enhanced-recovery pathway for primary total hip and knee arthroplasty. The three-phase roadmap comprised baseline pathway mapping and audit, pilot implementation and refinement, and full roll-out, punctuated by four multidisciplinary seminars. Key aspects of this programme included preoperative education, minimal fasting and early return to feeding, rational choice of regional anaesthesia techniques, administration of multimodal analgesia, reduction in urinary and surgical catheterization, active management of the risk of blood loss and deep vein thrombosis, optimisation of surgical workflow and techniques, and early mobilisation of patients. Global- and element-level compliance was tracked prospectively; ≥70% compliance was required for certification. External ERAS^®^ Society review at month 15 confirmed data integrity, sustainability planning, and successful certification. Continuous feedback loops drove micro-teaching and order-set optimisation throughout deployment phases. **Results**. Our ERAS programme for primary THA and TKA was introduced in April 2022. The definitive programme contained 24 mandatory elements grouped into three perioperative areas. After the fourth seminar, the rate of compliance was 81%. The certification was obtained in June 2023. **Conclusions**. Implementing an ERAS^®^ programme for primary total hip and knee arthroplasty is feasible within a high-volume academic institution when supported by multidisciplinary teamwork, robust data collection, and iterative feedback mechanisms. Further high-quality outcome-focused research is required to evaluate the clinical impact of individual ERAS components and to validate a personalised ERAS programme incorporating emerging technologies.

## 1. Introduction

Enhanced Recovery After Surgery (ERAS) is a structured, multidisciplinary programme designed to optimise the entire perioperative pathway through evidence-based, patient-centred, standardised care [1]. By combining preoperative preparation, optimised anaesthesia and analgesia, intraoperative optimisation with standardisation of the surgical workflow, minimally invasive techniques without tourniquet or drain, early mobilisation and targeted postoperative rehabilitation, the ERAS programme seeks to enhance functional recovery, shorten hospital stay and reduce complication rates, while simultaneously improving patient experience and satisfaction [1].

For total hip arthroplasty (THA) and total knee arthroplasty (TKA), the ERAS^®^ Society consensus statement published by Wainwright et al. provided a comprehensive set of recommendations that facilitate early ambulation, reduce medical and surgical complications, and increase patient-reported outcome scores [2]. Key elements include prehabilitation with strength improvement and mobility improvement; tailoring patient expectations on pain management and recovery time; preoperative optimisation, evidence-based fasting, standardised anaesthesia, blood sparing anaesthesia, surgical considerations, early postoperative care, and complication prevention.

Based on these recommendations and the contemporary literature, we have established an ERAS protocol for patients undergoing primary THA and TKA. The objective of this report is to determine whether it is feasible to implement an ERAS^®^ programme in orthopaedic surgery within our institution for primary THA and TKA.

## 2. Patients and Methods

### 2.1. Project Design and Governance

This work was conceived as a single-centre quality-improvement project that followed the Enhanced Recovery After Surgery (ERAS^®^) Society certification framework [2]. All activities were conducted in accordance with the Declaration of Helsinki and the hospital’s policy. After identifying a specific surgical procedure, a steering committee reporting to the orthopaedic departmental board provided strategic oversight. Day-to-day execution was delegated to an ERAS implementation team composed of a senior arthroplasty surgeon, a senior anaesthetist, an ERAS nurse coordinator, a physiotherapist, a ward nurse, and a data manager. The nurse coordinator scheduled meetings, maintained the protocol repository, and led compliance surveillance.

### 2.2. Step-Wise Implementation Process

The implementation roadmap contained four seminars and was structured in three phases, with the idea of developing an optimal patient-oriented procedure-specific care pathway. Throughout the implementation process, the ERAS team met regularly.

1. Seminar 1—Baseline mapping (Month 0).
  - Review of existing hip and knee perioperative pathways.
  - Retrospective audit of 100 consecutive primary THA/TKA cases to generate baseline metrics (length of stay, complication profile, element-level adherence).
2. Active phase 1 (Months 1–3).
  - Formation of procedure-specific working groups (hip and knee).
  - Drafting of evidence-based checklists using some of the ERAS^®^ Society orthopaedic consensus statement [2].
  - Identification of resource gaps (patient education material and electronic order sets).
3. Seminar 2—Protocol finalisation (Month 4).
  - Multidisciplinary review of draft elements.
  - Definition of hard-stop criteria for each ERAS item (e.g., “first mobilisation within 6 h” vs. “by postoperative day 1”).
4. Active phase 2 (Months 5–7).
  - Pilot implementation in 20 patients (10 THA and 10 TKA).
  - Rapid-cycle audit-and-feedback, used to refine wording, sequence, and responsibilities.
5. Seminar 3—Scale-up readiness (Month 8).
  - Presentation of pilot data, where an attainment of ≥70% global compliance threshold was required for certification [3].
  - Decision to deploy the method hospital wide.
6. Active phase 3 (Months 9–12).
  - Full roll-out to all primary elective THA/TKA cases.
  - Prospective data capture in a REDCap^TM^ registry, where automated dashboards are disseminated weekly.
7. Seminar 4—External audit and certification (Month 13).
  - On-site review by an ERAS^®^ Society coach.
  - Verification of compliance metrics, data integrity, and sustainability plan.
  - Formal certification achieved.

Compliance with each element was recorded as “achieved”, having a “justified deviation”, or being “missed”. Global compliance was calculated per patient (numerator: achieved elements, and denominator: applicable elements) and per element (proportion of patients in whom the element was achieved).

### 2.3. Hospital Stay and Postoperative Course

During their inpatient stay, patients track pain levels, functional milestones, and overall satisfaction in either a paper-based or digital logbook. This continuous self-monitoring fosters active engagement and empowerment, culminating in criteria-based discharge. Comprehensive home-support resources ensure patients leave with clear instructions on medications, wound care, red-flag symptoms, and physiotherapy—including prescribed self-directed exercises. After discharge, follow-up begins via the CHUV@Home digital platform, combining telemedicine consultations and automated reminders until the first outpatient visit 6 weeks post-surgery. The clinical pathway offers 24/7 access to a chat service staffed by nurses and physician assistants, complemented by real-time pop-up guidance, providing uninterrupted support and sustained patient involvement.

### 2.4. Data Management and Audit Strategy

Process-of-care metrics and protocol adherence data were entered contemporaneously by the ERAS nurse coordinator. Deviations triggered root-cause analysis and targeted counter-measures (micro-teaching, order-set adjustments). Monthly performance reports were submitted to the steering committee and were benchmarked against the ≥70% compliance target set after certification.

### 2.5. Ethical and Quality-Assurance Considerations

Because the initiative was categorised as service evaluation, formal research ethics committee approval and patient consent were not required under national regulations. Data were pseudonymised and stored on a secure institutional server; access was restricted to the ERAS team.

## 3. Results

Our ERAS programme for primary THA and TKA was introduced in April 2022. The definitive pathway contained 24 mandatory elements grouped into three perioperative areas (Table 1). After the fourth seminar, the rate of compliance was 81%. The certification was obtained in June 2023.

## 4. Discussion

After 15 months of iterative work, we successfully implemented an ERAS programme for primary THA and TKA that is closely aligned with the ERAS^®^ Society recommendations for arthroplasty [2]. The main deviation from the consensus statement concerns anaesthetic strategy, where we deliberately favoured neuraxial anaesthesia combined with selective peripheral nerve blocks over general anaesthesia and local infiltration.

Contemporary evidence consistently demonstrates that spinal anaesthesia is associated with lower early pain scores [4], reduced morbidity and mortality [5,6,7], shorter length of stay [5,6], and lower hospital costs [5,6] compared with general anaesthesia. Consequently, the ICAROS expert panel recommends neuraxial techniques for THA and TKA [8]. Our results confirm that high compliance with neuraxial anaesthesia is achievable in routine practice, without compromising theatre efficiency. Following a systematic review of 122 studies, the same panel also advocates adjunct peripheral nerve blocks to further enhance outcomes [9]. Consequently, we routinely add an adductor canal block combined with a selective tibial nerve block to address both anterior and posterior knee pain [10]. Local infiltration analgesia is an alternative [11]; however, the lack of agreement on optimal composition and technique [12] has led us to favour ultrasound-guided nerve blocks. For hip arthroplasty, our earlier work demonstrated that 100 µg intrathecal morphine alone affords excellent analgesia without the need for additional peripheral blocks [13].

Indeed, all patients received 100 µg of intrathecal morphine—shown to be the ceiling dose for analgesia and the threshold dose for postoperative nausea and vomiting [13]—as recommended by the PROSPECT group [14,15]. Contemporary original articles found no increased risk of sedation, respiratory depression, or hypoxaemia at this dose [16,17], supporting our practice of ward-level monitoring rather than 24 h high-dependency observation, as advocated by the ASA guidelines [18]. Indeed, these recommendations specify monitoring for a minimum of 24 h after administration (once per hour for the first 12 h after administration, followed by once every 2 h for the next 12 h, i.e., from 12 to 24 h). Based on the current evidence, we perceive these recommendations to be excessively cautious and are probably responsible for increased health resource consumption.

Consistent with ERAS principles, a multimodal opioid-sparing analgesic treatment should be started during the surgery and continued postoperatively to achieve optimal pain control [19,20]. Intraoperative dexamethasone (0.1–0.2 mg·kg^−1^) provided dual anti-emetic [21] and anti-inflammatory benefits without increasing perioperative hyperglycaemia, wound infection, or delayed healing [22,23,24]. Intravenous paracetamol, NSAIDs, and magnesium completed the multimodal regimen, enabling early mobilisation and reducing opioid consumption [25].

In our ERAS programme, 14 of the 17 implemented items relate directly to anaesthesia or peri-anaesthetic care. Importantly, these elements function as enablers for other core ERAS components, including early mobilisation, rapid return to oral nutrition, effective physiotherapy participation, and timely fulfilment of discharge criteria. Indeed, early pain control, haemodynamic stability, and minimisation of opioid-related adverse effects were prerequisites for successful mobilisation and functional recovery, underscoring the interdependence of anaesthetic, nursing, physiotherapy, and organisational domains rather than their isolation.

Intraoperative optimisation is central in ERAS protocols for hip and knee arthroplasty, and places stress on standard processes, minimal incisions, and the utilisation of technology. Standardisation of operating room workflows decreases variability in results and enables patients to more rapidly meet discharge criteria by making the process more efficient [20,26]. Although numerous analyses compared surgical methods—such as the direct anterior vs. posterolateral in hip arthroplasty—there is little convincing proof that one significantly outperforms the other within the ERAS environment [27]. In a large ERAS outpatient analysis, early discharge was attained employing standard posterior and medial parapatellar approaches in hip and knee replacements, respectively [28], indicating that consistency may be more important as a technique. Minimally invasive operations without the utilisation of a tourniquet or drain are increasingly popular, as the utilisation of tourniquets has been correlated with slower recovery, more discomfort, and greater thrombotic and wound risk, despite failing to discernibly lessen blood loss or cement quality [29,30]. Similarly, routine utilisation of surgical drains in ERAS is increasingly challenged, as there is strong evidence that they do not prevent complications such as haematoma or infection and may even contribute to worse blood loss and allogenic transfusion requirements; utilisation is thus in conflict with ERAS aims at rapid recovery and minimal interference [31]. Their exclusion in proved ERAS programmes in the absence of resultant complications lends this approach credibility [32]. Surgery with robotics has the promise for greater precision, minimal invasiveness, preservation of soft tissue, and reduced learning curve for surgeons, though studies have not shown it in isolation to enable earlier discharge. The lack of clear superiority of one technique underscores the importance of consistency and team coordination brought by ERAS implementation rather than innovation alone. Ultimately, while intraoperative elements are crucial, such as the duration of surgery [33], no single factor independently guarantees accelerated recovery; the cumulative effect within a standardised, minimally invasive, and team-based ERAS programme remains paramount.

In the future, personalisation of the ERAS programme is the logical next step. Predictive analytics and patient profiling may help tailor interventions and optimise resource allocation. Technological advances—robot-assisted surgery, wearable sensors, and tele-rehabilitation—could further enhance functional recovery and shorten length of stay. Moreover, it should increasingly be leveraged to expand the proportion of cases performed in an ambulatory setting [34,35,36], as complication rates are comparable to those observed in traditional in-hospital surgery [37,38]. Building on the experience gained with primary arthroplasty, we have begun extending ERAS protocols to revision THA/TKA, shoulder arthroplasty, and rotator-cuff repair.

This study has several limitations. First, this ERAS^®^ programme was implemented in a high-volume, well-resourced academic centre with established multidisciplinary collaboration and dedicated digital infrastructure, which may limit direct transferability to smaller or less-resourced institutions. Adaptation of this model in other settings may require simplification of pathways, staged implementation, or alternative data-capture strategies tailored to local resources and organisational structures. Second, the design was descriptive and focused on implementation, without a formal control group or statistical comparison of outcomes; as such, causality cannot be inferred. Third, the follow-up period was relatively short and centred on achieving certification, meaning that long-term sustainability of compliance and patient outcomes remains to be evaluated. Then, although overall ERAS^®^ programme compliance was prospectively assessed, compliance with individual ERAS elements was not analysed in detail. As a result, variability in adherence across specific components of the programme could not be evaluated in the present report. Moreover, compliance with ERAS^®^ elements was prospectively recorded by the implementation team, which may introduce observer or confirmation bias, despite adherence to the ERAS^®^ Society certification framework. Finally, although we tracked compliance and key perioperative parameters, we did not assess broader patient-reported outcomes such as quality of life, satisfaction, or functional recovery beyond the early postoperative phase. These factors should be addressed in future studies to strengthen the evidence base for ERAS^®^ implementation in orthopaedic surgery.

## 5. Conclusions

Implementing an ERAS^®^ programme for primary total hip and knee arthroplasty is feasible within a high-volume academic institution when supported by multidisciplinary teamwork, robust data collection, and iterative feedback mechanisms. This implementation study demonstrates successful pathway development, high compliance with ERAS elements, and achievement of the ERAS^®^ Society certification. Further high-quality outcome-focused research is required to evaluate the clinical impact of individual ERAS components and to validate a personalised ERAS programme incorporating emerging technologies.

## Figures and Tables

**Table 1:** Total hip (THA) and knee (TKA) arthroplasty pathway.

Preoperative (7 items)
Structured patient education programme and take–home booklet: counselling, informing patients about the procedure, recovery timeline, and self-care responsibilities
Risk stratification and medical optimisation, including management of comorbidities
Anaemia screening and correction (intravenous iron ± erythropoietin if haemoglobin < 130 g·L^−1^)
Venous thrombo-embolism risk stratification and plan
Kondrup Nutritional Risk Screening—2002 (≥3 triggers dietitian referral)
Prehabilitation (exercise and nutritional support to build strength and stamina)
Preoperative fasting: solids up to 6 h and clear fluids up to 2 h before induction, with routine carbohydrate loading (200 mL 12.5%) up to 2 h preoperatively
Intraoperative (10 items)
Standardised regional anaesthesia: no premedication, spinal anaesthesia with bupivacaine and low-dose intrathecal morphine (100 µg), along with peripheral nerve block (adductor canal and selective tibial nerve blocks for patients undergoing TKA, with no blocks for patients undergoing THA)
Opioid-sparing multimodal analgesia: intravenous dexamethasone (0.1–0.2 mg·kg^−1^), paracetamol (1 g), ketorolac (30 mg), and magnesium (40–50 mg·kg^−1^)
Postoperative nausea and vomiting prophylaxis: dexamethasone and ondansetron
Light sedation with propofol on request
Normothermia maintenance: forced-air warming system and warmed fluids
Blood-sparing strategies: single-dose intravenous tranexamic acid (15 mg·kg^−1^) before incision, restrictive transfusion thresholds, and meticulous haemostasis
Minimally invasive approach with standardisation of the surgical workflow and robotisation of the procedures for total knee arthroplasty, without tourniquet and surgical drains, to reduce soft tissue trauma
Perioperative fluid management: restrictive fluid administration and maintenance of euvolemia
No routine urinary catheter
Standardised antibiotic prophylaxis (cefuroxime 1.5 g 30–60 min before incision)
Postoperative (7 items)
Early return to oral fluid intake and normal diet, and early mobilisation: “Drink–Eat–Mobilise” paradigm on the day of surgery (clear fluids within 2 h, oral intake within 4 h, and first assisted walk within 6 h)
Structured physiotherapy beginning on postoperative day 0–1 with functional objectives (sitting, standing, and walking with assistance, stairs)
Multimodal analgesia: paracetamol, NSAID unless contraindicated, and patient-controlled oral opioid if pain score > 4/10)
Early removal (within 24 h) of intravenous lines and urinary catheter (if inserted in case of postoperative urinary retention)
Venous thrombo-embolism prophylaxis with low molecular weight heparin from 6 h postoperatively, followed by direct oral anticoagulants for 35 days
Hospital discharge criteria: adequate pain control with standard oral analgesics, independent ambulation with full weight-bearing using two crutches over at least 100 m, independent stair negotiation, and knee flexion ≥ 90° following total knee arthroplasty
Post-hospitalisation follow-up (nurse, physiotherapist and surgeon)

NSAID, non-steroidal anti-inflammatory drug.

## Data Availability

No new data were created or analysed in this study.
